# Linear Ubiquitin Chains: Cellular Functions and Strategies for Detection and Quantification

**DOI:** 10.3389/fchem.2019.00915

**Published:** 2020-01-10

**Authors:** Gunnar Dittmar, Konstanze F. Winklhofer

**Affiliations:** ^1^Proteomics of Cellular Signalling, Quantitative Biology Unit, Luxembourg Institute of Health, Strassen, Luxembourg; ^2^Department of Molecular Cell Biology, Institute of Biochemistry and Pathobiochemistry, Ruhr University Bochum, Bochum, Germany

**Keywords:** ubiquitin, HOIP, HOIL, SHARPIN, LUBAC, OTULIN, SRM, PRM

## Abstract

Ubiquitination of proteins is a sophisticated post-translational modification implicated in the regulation of an ever-growing abundance of cellular processes. Recent insights into different layers of complexity have shaped the concept of the ubiquitin code. Key players in determining this code are the number of ubiquitin moieties attached to a substrate, the architecture of polyubiquitin chains, and post-translational modifications of ubiquitin itself. Ubiquitination can induce conformational changes of substrates and alter their interactive profile, resulting in the formation of signaling complexes. Here we focus on a distinct type of ubiquitination that is characterized by an inter-ubiquitin linkage through the N-terminal methionine, called M1-linked or linear ubiquitination. Formation, recognition, and disassembly of linear ubiquitin chains are highly specific processes that are implicated in immune signaling, cell death regulation and protein quality control. Consistent with their role in influencing signaling events, linear ubiquitin chains are formed in a transient and spatially regulated manner, making their detection and quantification challenging.

## Introduction

**Figure 1:**
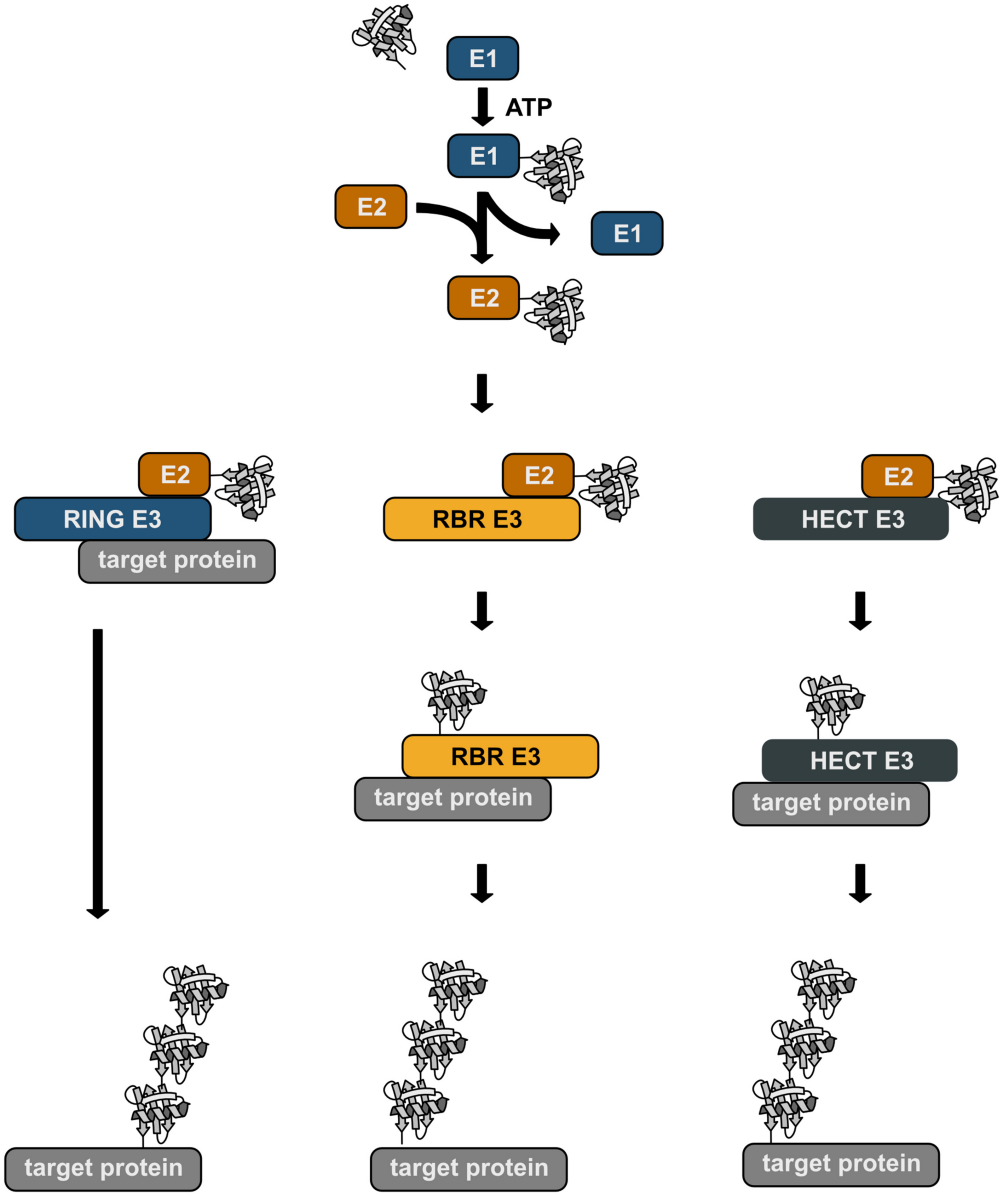
Enzymatic cascade of ubiquitination. Ubiquitin is transferred to the target protein by an enzymatic cascade. Ubiquitin is first bound by an ubiquitin-activating enzyme (E1) using one ATP molecule. The activated ubiquitin is then transferred to a conjugating enzyme (E2). Depending on the type of the E3 ubiquitin ligase that is involved in the ubiquitination process, ubiquitin is directly transferred from the E2 to the target protein with the ligase acting as specific bridging factor (RING ligases). Alternatively, the ubiquitin moiety is transferred to the E3 ligase (RBR and HECT ligases) via a transient thioester bond before it is attached to the target protein by an isopeptide bond.

Ubiquitination is a reversible post-translational modification that can affect the function, the fate, and the subcellular localization of the modified substrates, thereby regulating fundamental cellular processes (Akutsu et al., 2016; Swatek and Komander, 2016; Yau and Rape, 2016). The transfer of ubiquitin is catalyzed by an enzymatic cascade involving three enzymes, an E1 ubiquitin-activating enzyme, an E2 ubiquitin-conjugating enzyme, and an E3 ubiquitin ligase. E3 ubiquitin ligases fall into three categories: RING/U-box ligases, RBR (RING-between-RING) ligases, and HECT ligases. The mechanisms of ubiquitin transfer to the target protein varies depending on the E3 ligase type (Figure 1). RING ligases facilitate the direct transfer of ubiquitin from a ubiquitin-charged E2 to the substrate. HECT ligases directly bind ubiquitin by forming a thioester intermediate via a catalytic cysteine residue. From this thioester ubiquitin is passed on to a lysine residue of the substrate, generating an isopeptide bond. RBR ligases use a RING/HECT hybrid mechanism. Similarly to RING ligases, they bind an E2 ubiquitin-conjugating enzyme via their RING1 domain. Ubiquitin is then transferred from the E2 to a catalytic cysteine in the RING2 domain forming a transient thioester, similarly to HECT ligases. This ubiquitin moiety is then attached to the target protein.

**Figure 2:**
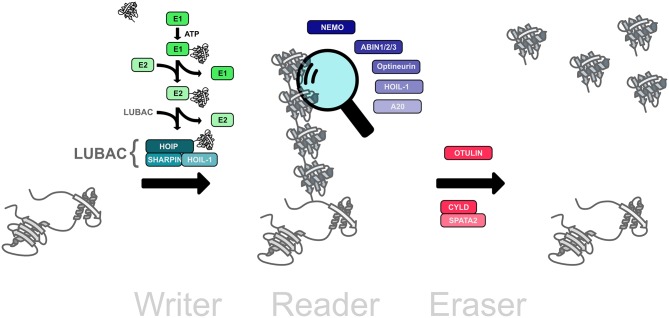
The linear ubiquitination machinery. Linear ubiquitin chains are assembled by LUBAC, the linear ubiquitin chain assembly complex, comprising HOIL, HOIL-1, and SHARPIN (writers). This modification can be translated into a cellular effect by proteins that specifically interact with linear ubiquitin chains (readers). The linear ubiquitination signal can be removed by deubiquitinases that disassemble M1-linked ubiquitin chains (erasers).

The ubiquitination machinery requires not only proteins to create the ubiquitin modifications, but also proteins to recognize and remove ubiquitin moieties (Figure 2). The ubiquitin signal is decoded and thereby translated into cellular effects by proteins harboring one or several ubiquitin-binding domains (UBDs), some of which recognize ubiquitin chain topologies with high selectivity (Dikic et al., 2009; Fennell et al., 2018). Reversibility of ubiquitination is ensured by deubiquitinases that hydrolyze isopeptide or peptide bonds between ubiquitin molecules or between ubiquitin and substrate proteins (Dikic et al., 2009; Mevissen and Komander, 2017; Fennell et al., 2018).

**Figure 3:**
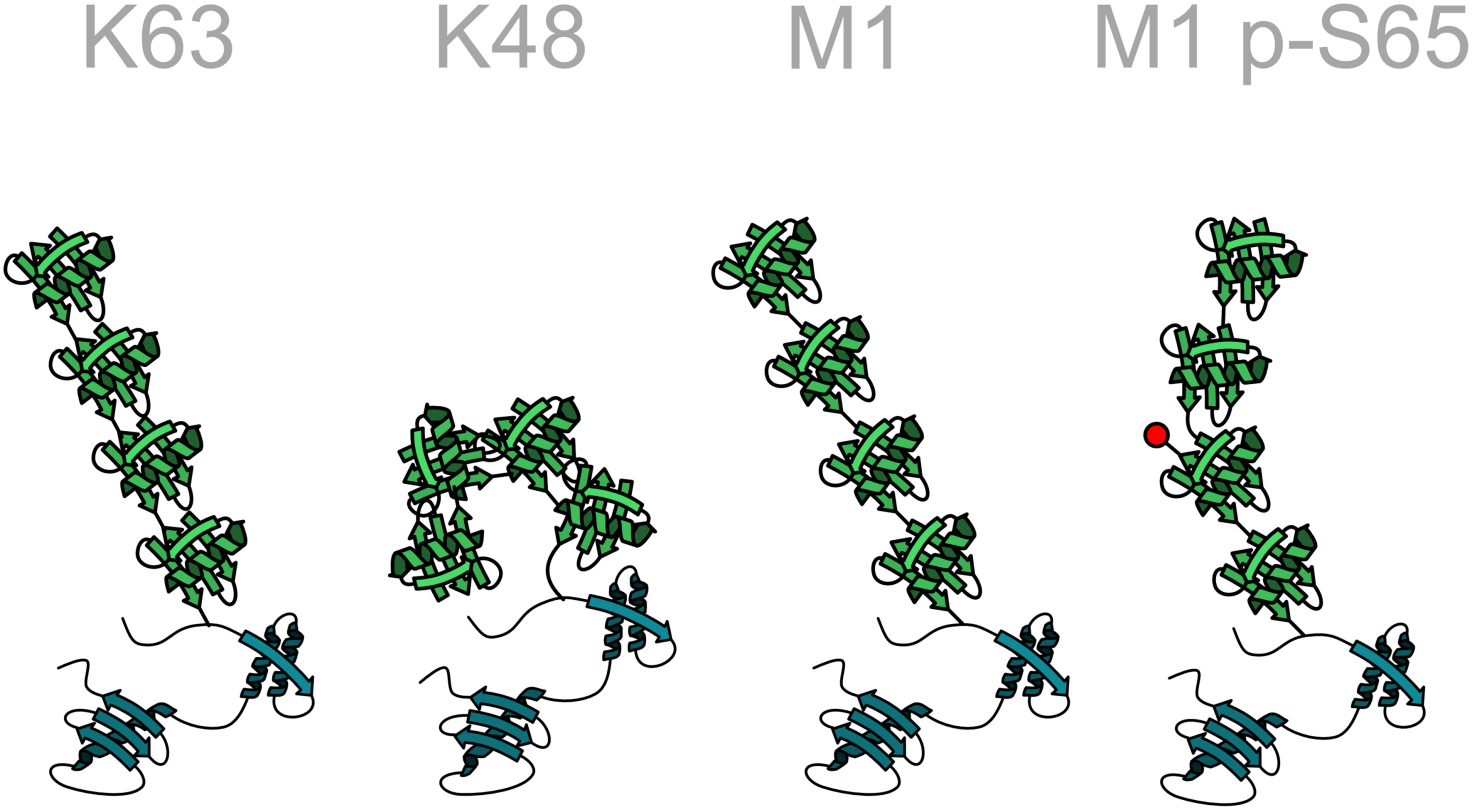
Structural diversity of ubiquitin chains. In homotypic ubiquitin chains ubiquitin monomers are linked to one of seven lysine (K) residues or to the N-terminal methionine (M1). For example, in a K48-linked ubiquitin chain all ubiquitin monomers are linked to lysine 48 of the acceptor ubiquitin moiety. Different linkage types are characterized by specific conformations of the polyubiquitin chain, such as an open or closed conformation. The structure of the chain can be further modified by post-translational modifications of ubiquitin, here indicated by the phosphorylation of serine 65 in a linear ubiquitin chain. Ubiquitin: green; target protein: blue.

Ubiquitination is the most versatile post-translational modification based on variabilities in the number of ubiquitin moieties attached to a substrate, the mode of inter-ubiquitin linkage, and the formation of heterotypic (mixed or branched) ubiquitin chains. Substrate proteins can be modified with single ubiquitin moieties or with polymeric ubiquitin chains. Within polyubiquitin chains, ubiquitin can form eight different linkage types, using one of seven internal lysine residues (K6, K11, K27, K29, K33, K48, K63) or methionine at position 1 (M1). Additional layers of complexity emerge from the formation of heterotypic chains with mixed linkages, branched chains, and the post-translational modification of ubiquitin itself by phosphorylation, acetylation, sumoylation, and neddylation, reminiscent of a sophisticated and highly versatile code. Each linkage-type has a distinct three-dimensional topology allowing interactions with linkage-specific effector proteins, thus resulting in specific biological outcomes (Figure 3).

## The Linear Ubiquitination Machinery

**Figure 4:**
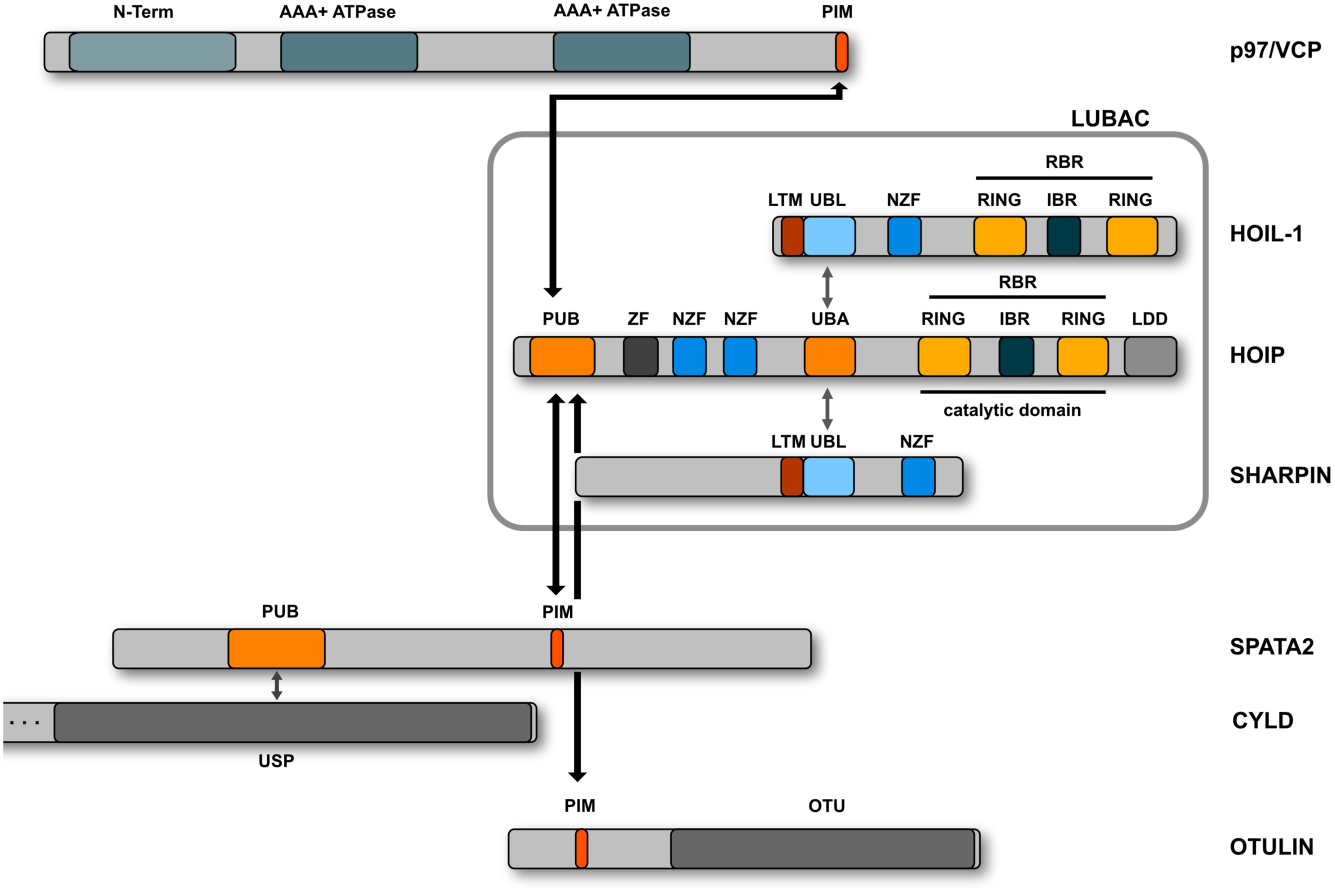
Domain structure of the LUBAC components. LUBAC consists of three core proteins, HOIP, HOIL-1, and SHARPIN. HOIP and HOIL-1 are RBR E3 ligases characterized by a RING - IBR (in between RING) - RING domain structure. The interaction between the UBL domains of HOIL-1 and SHARPIN with the UBA domain of HOIP releases the autoinhibition of HOIP. HOIP interacts via its PUB domain with the PIM domain of OTULIN, SPATA2, or p97/VCP.

M1-linked or linear ubiquitination is characterized by the head-to-tail linkage of ubiquitin molecules via the C-terminal carboxyl group of the donor ubiquitin and the N-terminal methionine of the acceptor ubiquitin. This results in the formation of a peptide bond in contrast to isopeptide formation via the linkage to the epsilon amino group of a lysine residue. The M1 linkage is generated by the linear ubiquitin chain assembly complex (LUBAC) that has first been described in 2006 as a complex of about 600 kDa containing the two RBR E3 ubiquitin ligases HOIP and HOIL-1 (Kirisako et al., 2006). Some years later, the adaptor protein SHARPIN was identified as the third core component of LUBAC (Gerlach et al., 2011; Ikeda et al., 2011; Tokunaga et al., 2011) (Figure 4). HOIP is the catalytically active component of LUBAC and the only E3 ubiquitin ligase that can assemble M1-linked ubiquitin based on its unique C-terminal linear ubiquitin chain determining domain (LDD) that positions the N-terminus of the target ubiquitin (Smit et al., 2012; Stieglitz et al., 2012; Yagi et al., 2012; Lechtenberg et al., 2016; Liu et al., 2017). The catalytic activity of HOIP is autoinhibited by its N-terminal domain so that full-length HOIP has no linear ubiquitination activity *in vitro*, in contrast to the N-terminally truncated RBR-LDD domain (Smit et al., 2012; Stieglitz et al., 2012, 2013; Yagi et al., 2012; Liu et al., 2017; Fujita et al., 2018). Binding of the ubiquitin-like (UBL) domain of HOIL-1 and SHARPIN to the ubiquitin-associated domain (UBA) of HOIP releases HOIP from autoinhibition (Smit et al., 2012; Stieglitz et al., 2012, 2013; Liu et al., 2017; Fujita et al., 2018). *In vitro*, the HOIL-1 UBL domain and the SHARPIN UBL domain can separately or synergistically bind to different regions within the UBA domain of HOIP (Liu et al., 2017). Both UBLs can induce conformational changes in the HOIP UBA domain, which allosterically rearrange the orientation between the UBA and RBR-LDD, facilitating E2 loading and promoting catalytic activity of HOIP (Liu et al., 2017). The crystal structure of the trimeric LUBAC core revealed that HOIL-1 and SHARPIN interact with each other via LUBAC-tethering motifs (LTMs) located N-terminally to the UBL domains of both proteins (Fujita et al., 2018). Upon heterodimerization, both LTMs fold into a single globular domain that plays a critical role in stabilizing trimeric LUBAC (Fujita et al., 2018).

HOIL-1 apparently has also catalytic activity. It has been reported to undergo auto-ubiquitination (Tatematsu et al., 2008) and to ubiquitinate oxidized IRP2 (iron regulatory protein 2), thereby inducing IRP2 degradation (Yamanaka et al., 2003). Recombinant HOIL-1 only lacking a C-terminal tail of 32 amino acids was observed to generate high molecular weight ubiquitin chains albeit with low efficiency (Stieglitz et al., 2012; Liu et al., 2017; Fujita et al., 2018). In addition to activating the catalytic core of HOIP, HOIL-1 obviously helps to direct the first ubiquitin toward a lysine residue of the substrate (Smit et al., 2013). The RBR-LDD domain of HOIP does assemble free linear ubiquitin chains *in vitro* but does not modify NEMO (NF-κB essential modifier), a key LUBAC substrate. However, in the presence of catalytically active HOIL-1, linear ubiquitin chain formation at NEMO lysines is efficient (Smit et al., 2013). The assembly of linear ubiquitin chains on substrates by HOIP requires priming of the first ubiquitin on a substrate lysine residue followed by the linkage of an incoming ubiquitin to the N-terminus of the “primed” target ubiquitin. HOIP assembles linear ubiquitin chains preferentially on K63-ubiquitinated substrates, resulting in heterotypic ubiquitin chains (Emmerich et al., 2013, 2016; Fiil et al., 2013; Hrdinka et al., 2016). In support of this notion, the RBR E3 ubiquitin ligase Parkin can increase LUBAC-mediated linear ubiquitination of NEMO by modifying NEMO with K63-linked ubiquitin (Henn et al., 2007; Sha et al., 2010; Müller-Rischart et al., 2013; Asaoka et al., 2016).

Recently, HOIL-1 was found to act as an atypical E3 ligase by forming an oxyester bond between the C-terminus of ubiquitin and serine or threonine residues (Kelsall et al., 2019). This activity of HOIL-1 is implicated in its auto-ubiquitination and in the modification of substrates within Toll-like receptor signaling, such as IRAK1, IRAK2, and MyD88, by monoubiquitin (Kelsall et al., 2019). Monoubiquitin attached to substrates by HOIL-1 via an oxyester bond can act as a target for further ubiquitination, suggesting a role of HOIL-1 in initiating polyubiquitin chain formation.

Several proteins have been described to interact with linear ubiquitin chains via specific ubiquitin-binding domains (UBDs) (reviewed in Fennell et al., 2018; Figure 2). These interactors include proteins with a UBAN (UBD in ABIN proteins and NEMO) domain, such as NEMO, ABIN-1, ABIN-2, ABIN-3, and Optineurin. HOIL-1 and A20 interact via zinc finger domains with M1-linked ubiquitin. In addition, the deubiquitinases OTULIN and CYLD, which both are capable of hydrolyzing M1-linked polyubiquitin, bind to linear ubiquitin chains through their catalytic domains.

OTULIN is the only known deubiquitinase that exclusively disassembles linear ubiquitin chains (Keusekotten et al., 2013; Rivkin et al., 2013). The reason for this specificity is based on two features: First, OTULIN binds with high affinity to M1-linked polyubiquitin and second, it employs a mechanism of ubiquitin-assisted catalysis, implicating activation of the catalytic triad by the proximal ubiquitin moiety (Keusekotten et al., 2013). OTULIN binds to the N-terminal PUB (PNGase/UBA or UBX-containing proteins) domain of HOIP via its PUB-interacting motif (PIM) and this interaction seems to be regulated by phosphorylation (Elliott et al., 2014; Schaeffer et al., 2014; Takiuchi et al., 2014). The PUB domain of HOIP can also interact with SPATA2 that binds CYLD and thereby bridges this deubiquitinase to LUBAC (Elliott et al., 2016; Kupka et al., 2016; Schlicher et al., 2016; Wagner et al., 2016). CYLD hydrolyzes both K63- and M1-linked ubiquitin chains (Komander et al., 2009; Sato et al., 2011; Ritorto et al., 2014) and together with OTULIN regulates signaling by linear ubiquitin chains. In contrast to CYLD, OTULIN prevents LUBAC from auto-ubiquitination (Fiil et al., 2013; Keusekotten et al., 2013; Hrdinka et al., 2016; Heger et al., 2018). Importantly, binding of OTULIN and SPATA2 to HOIP is mutually exclusive, since both proteins compete for binding to the PUB domain (Draber et al., 2015; Elliott et al., 2016). Whereas the absence of OTULIN induces a strong increase in the abundance of M1-linked ubiquitin (Rivkin et al., 2013; Damgaard et al., 2016), this is not observed in the absence of CYLD (Draber et al., 2015). It is therefore conceivable that CYLD exerts a ubiquitin chain-editing function by trimming K63-linked chains and influencing K63-M1-hybrid chain formation (Emmerich et al., 2013, 2016; Hrdinka et al., 2016).

## Cellular Functions of Linear Ubiquitin Chains

### LUBAC and TNF Signaling

**Figure 5:**
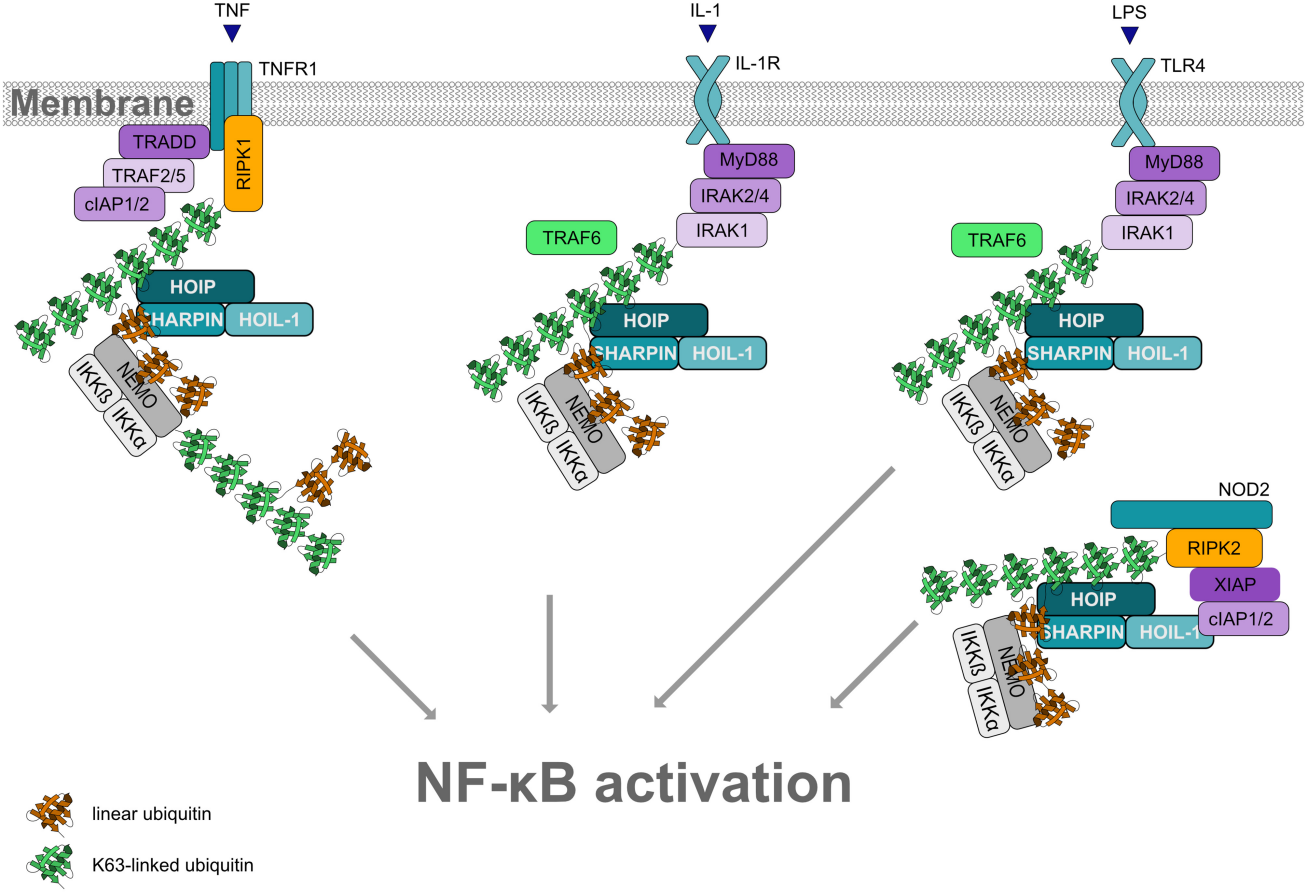
Linear ubiquitination and NF-κB activation. Formation of linear ubiquitin chains is implicated in NF-κB activation induced by different pathways, for example via TNF or IL-1 receptors, Toll-like receptors or NOD2 receptors. All these pathways involve the formation of signaling complexes to which LUBAC is recruited via K63-linked ubiquitin. K63-linked polyubiquitin is generated by cIAP1/2 at the TNFR complex and by TRAF6 at the IL-1R and at TLRs. LUBAC then assembles M1-linked ubiquitin on K63-ubiquitinated NEMO (and other substrates within the pathway). Oligomerization of NEMO activates the associated kinases IKKα and IKKβ, required for NF-κB activation.

Linear ubiquitin chains generated by LUBAC play a key role in regulating innate and adaptive immunity and inflammatory signaling, for example via the TNF receptor (TNFR1), IL-1 receptor, CD40, TRAIL receptor, Toll-like receptors (TLRs), T and B cell receptors, NOD1 and NOD2 receptors, RIG-I receptors, and the NLRP3 inflammasome (reviewed in Iwai et al., 2014; Hrdinka and Gyrd-Hansen, 2017; Rittinger and Ikeda, 2017; Spit et al., 2019; Figure 5). Consistent with the regulation of these pathways by M1-linked ubiquitin, several LUBAC substrates have been identified, such as NEMO, RIPK1, RIPK2, TRADD, TNFR1, IRAK1/2/4, and MyD88 (Haas et al., 2009; Tokunaga et al., 2009; Gerlach et al., 2011; Emmerich et al., 2013; Fiil et al., 2013; Draber et al., 2015; Wertz et al., 2015; Kelsall et al., 2019).

LUBAC function has most widely been studied in the context of TNF signaling (reviewed in Peltzer and Walczak, 2019; Spit et al., 2019). In 2009, LUBAC was shown to activate canonical NF-κB signaling in response to TNF or IL-1 stimulation by conjugating linear ubiquitin chains on NEMO, the core regulatory component of the IκB kinases (IKK) complex (Haas et al., 2009; Tokunaga et al., 2009). Moreover, NEMO harbors a UBAN domain that binds to M1-linked ubiquitin with high affinity (Komander et al., 2009; Rahighi et al., 2009). Upon binding of TNF to its receptor at the plasma membrane, a multiprotein signaling complex, denoted complex I, is assembled, which is regulated by phosphorylation and ubiquitination. K63-linked polyubiquitin generated by cIAP1/2 recruits LUBAC to the activated TNFR1. LUBAC adds linear ubiquitin chains to various substrates, resulting in the formation of mixed K63-/M1-linked heterotypic chains. Since NEMO is not only modified by M1-linked ubiquitin, but also binds to M1-linked ubiquitin via its UBAN domain, linear ubiquitination of NEMO promotes its oligomerization. This induces a conformational change of the associated kinases IKKα and IKKβ within the IKK complex, leading to their activation. Activated IKKs phosphorylate the NF-κB inhibitor IkBα, which is subsequently modified with K48-linked ubiquitin and degraded by the proteasome. Thus, NF-κB heterodimers are released from their inhibitory binding and translocate into the nucleus to regulate the expression of NF-κB target genes. Depending on the cell type and cellular context, NF-κB upregulates pro-survival and/or inflammatory gene expression.

When the formation of the cytoprotective complex I is compromised, for example through defective ubiquitination mediated by either cIAPs or LUBAC, complex II is generated that induces cell death (reviewed in Dondelinger et al., 2016). A crucial player in the transition between complex I and complex II is RIPK1. Phosphorylation of RIPK1 at specific sites for example by TAK1, IKKα, IKKβ, IKKε, or TBK1 has been shown to prevent complex II formation by keeping RIPK1 in an inactive, non-autophosphorylated state (Dondelinger et al., 2015; Annibaldi and Meier, 2018; Lafont et al., 2018; Xu et al., 2018). Notably, TBK1 and IKKε are recruited to complex I mostly by M1-linked ubiquitin (Lafont et al., 2018). Complex II can promote either apoptosis (when caspase-8 is active) or necroptosis, induced by the necrosome formed by RIPK1, RIPK3, and MLKL (reviewed in Peltzer and Walczak, 2019).

### LUBAC-Associated Pathologies

In support of a substantial role of LUBAC in regulating immune signaling, mice deficient in the expression of LUBAC components suffer from severe phenotypes. Both HOIP knockout (KO) and HOIL-1 KO mice lacking the UBL domain are not viable and die around E10.5 (Emmerich et al., 2013; Sasaki et al., 2013; Peltzer et al., 2014; Fujita et al., 2018). Interestingly, mice expressing catalytically inactive HOIL-1 (C458S knock-in mice) are viable, since this mutant can still bind and stabilize HOIP in contrast to HOIL-1 lacking the UBL domain (Kelsall et al., 2019). Mice with a spontaneous autosomal recessive loss-of-function mutation in the SHARPIN gene develop chronic proliferative dermatitis, systemic inflammation, and increased apoptosis in the liver, lung, and skin (Seymour et al., 2007; Gerlach et al., 2011; Ikeda et al., 2011; Tokunaga et al., 2011). Notably, defective disassembly of M1-linked ubiquitin has also severe consequences. Mice homozygous for missense mutations interfering with OTULIN function, exhibit embryonic lethality between E12.5 and E14, characterized by vascularization defects and impaired Wnt signaling (Rivkin et al., 2013).

In humans, reduced HOIP expression due to a missense mutation in the HOIP gene causes multiorgan autoinflammation and immunodeficiency (Boisson et al., 2015). These clinical phenotypes widely overlap with those seen in some HOIL-1-deficient patients. Depending on the type of mutation, these patients show autoinflammation and immunodeficiency or polyglucosan storage myopathy (muscular amylopectinosis) and cardiomyopathy (Boisson et al., 2012; Nilsson et al., 2013; Wang et al., 2013). Interestingly, homozygous loss-of-function mutations in the OTULIN gene cause an auto-inflammatory condition, called ORAS (OTULIN-related inflammatory syndrome) or otulipenia that is responsive to anti-TNF treatment (Damgaard et al., 2016; Zhou et al., 2016). These multifaceted pathologies underpin the complex interplay between assembly and disassembly of linear ubiquitin chains, requiring tight regulation and fine-tuned balancing in a cell-type- and context-specific manner.

Given its role in cell death regulation, LUBAC is also associated with oncogenic signaling. Two germline missense single nucleotide polymorphisms (SNPs) in the gene encoding HOIP are enriched in patients suffering from a subtype of diffuse large B cell lymphoma (DLBCL). In activated B cell-like (ABC) DLBCL the constitutive activation of NF-κB mediated by B cell receptor signaling (implicating CARD11, MALT1, and BCL10) and MyD88 signaling is a major pathogenic mechanism, promoting malignant cell survival. The two SNPs identified in ABC DLBCL are located in the UBA domain of HOIP affecting the HOIP/HOIL-1 interface and were shown to enhance LUBAC activity and NF-κB signaling (Yang et al., 2014). In addition, oncogenic CARD11 mutants found in ABC DLBCL spontaneously induce linear ubiquitination of BCL10 by enhancing the interaction between HOIP and BCL10 (Yang et al., 2016b). LUBAC recruitment to BCL10 is promoted by cIAP1/2 which assemble K63-linked ubiquitin chains on BCL10 and on themselves (Yang et al., 2016a). In an siRNA screen, HOIP was identified as a modifier of cisplatin-induced toxicity (MacKay et al., 2014). Depletion of HOIP or expression of catalytically inactive HOIP sensitizes different cancer cell lines to genotoxin-induced apoptotic cell death. Supporting a role of LUBAC in chemotherapy resistance, expression of LUBAC components is significantly higher in cisplatin-resistant cancer cell lines (MacKay et al., 2014) and patient samples and preclinical mouse models of lung squamous cell carcinoma (LSCC) (Ruiz et al., 2019). Moreover, the small molecule HOIP inhibitor gliotoxin sensitizes LSCC cells and mice to cisplatin (Ruiz et al., 2019). A recent study linked LUBAC to chromosome alignment during mitosis. LUBAC was reported to ubiquitinate the kinetochore motor CENP-E that binds in its M1-ubiquitinated form to the linear ubiquitin chain receptor KLN1 (kinetochore null protein 1) at attached kinetochors thereby promoting accurate chromosome segregation (Wu et al., 2019). Whether LUBAC is a feasible drug target to treat malignant diseases needs to be explored in future studies.

### LUBAC and Intracellular Bacteria

It has recently been discovered that LUBAC is recruited to the surface of cytosol-invading bacteria, such as *Salmonella enterica* serovar Typhimurium, that have escaped the endocytic pathway and therefore are no longer shielded by host membranes (Zhou et al., 2016; Noad et al., 2017; van Wijk et al., 2017). Bacterial surface components as well as associated host membrane remnants are ubiquitinated by several E3 ubiquitin ligases to generate a ubiquitin coat (Perrin et al., 2004). HOIP binds to this ubiquitin coat via its N-terminal NZF domains (Noad et al., 2017). In addition, the catalytic activity of HOIP is required for its recruitment, suggesting a feed-forward mechanism through synthesizing and binding M1-linked ubiquitin at the bacterial surface (Noad et al., 2017). Linear ubiquitin chains generated by LUBAC recruit the effector proteins NEMO and Optineurin to cytosolic bacteria. As a consequence, two events are induced that independently restrict bacterial proliferation: Local activation of NF-κB mediated by NEMO and stimulation of antibacterial autophagy (xenophagy) mediated by Optineurin (Noad et al., 2017). These effects can be enhanced by decreasing the expression of OTULIN via RNA interference or CRISPR/Cas9 knockout (van Wijk et al., 2012; Noad et al., 2017).

### LUBAC and Protein Quality Control

**Figure 6:**
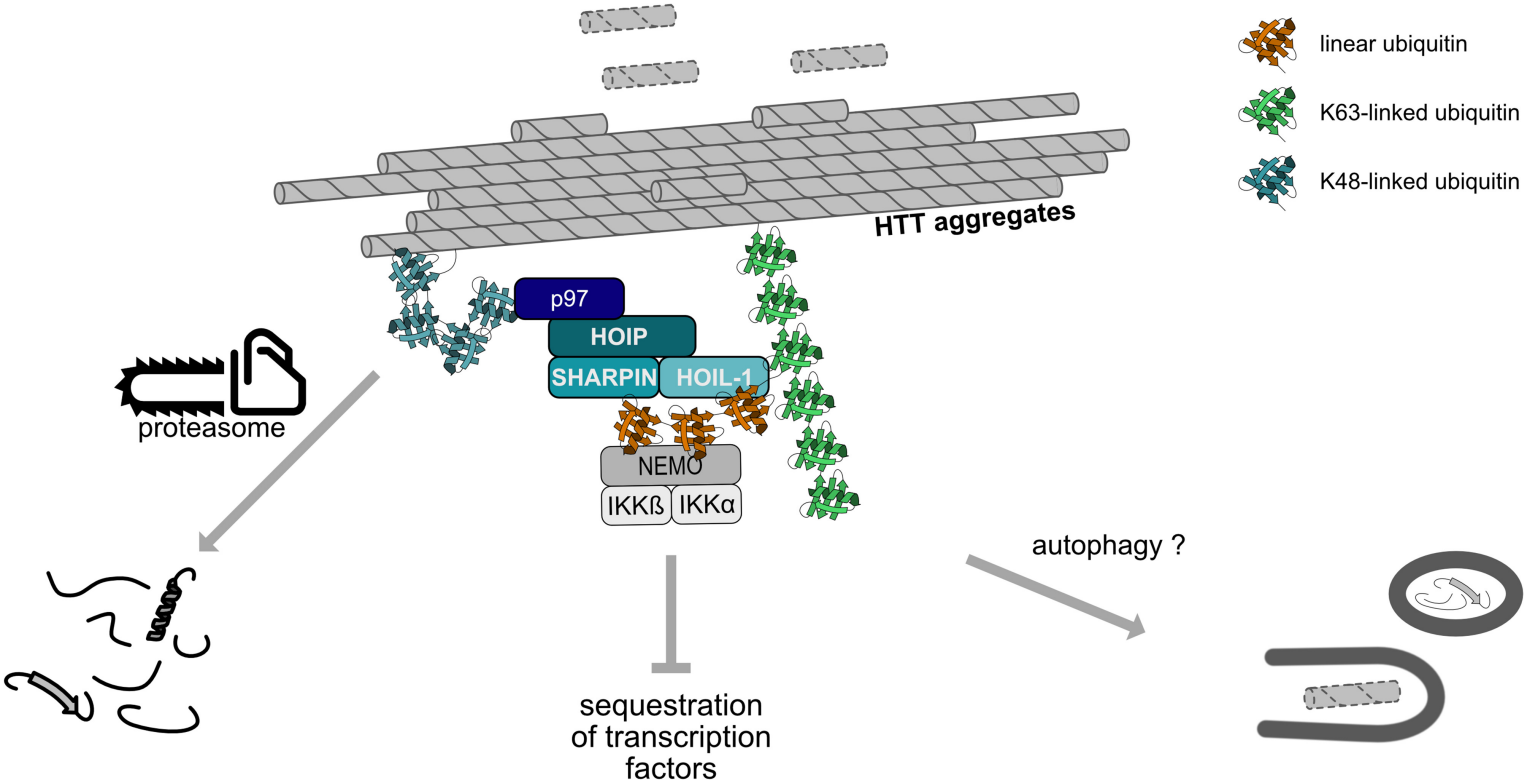
Linear ubiquitination of Huntingtin aggregates. Huntingtin (Htt) aggregates are covered by a ubiquitin coat, including K48- and K63-linked chains. HOIP is recruited to these aggregates in a p97/VCP-dependent manner and together with HOIL-1 and SHARPIN assembles M1-linked ubiquitin chains. Subsequent recruitment of proteins specifically interacting with linear ubiquitin, such as NEMO, remodels the interactive surface of the aggregates. Linear ubiquitination promotes proteasomal degradation of misfolded Htt species and may also increase the removal of aggregates by autophagy.

LUBAC is not only recruited to cytosolic bacteria but also to cytosolic protein aggregates, suggesting that assemblies of misfolded proteins are sensed as a special kind of “cellular pathogen” or danger-associated molecular pattern (van Well et al., 2019). We observed that LUBAC modifies misfolded Huntingtin containing a pathogenic polyglutamine expansion (Htt-polyQ) with M1-linked ubiquitin and thereby shapes the ubiquitin coat of these aggregates. Linear ubiquitination of both cytosolic bacteria and aggregates has beneficial cellular effects, yet mediated by different mechanisms (Figure 6). HOIP is recruited to protein aggregates by p97/VCP, a triple A-type quality control ATPase that can extract ubiquitinated proteins from macromolecular complexes or lipid membranes. p97/VCP also has a PIM domain which is required for the interaction with the PUB domain of HOIP (Elliott et al., 2014; Schaeffer et al., 2014; Takiuchi et al., 2014). As a consequence of linear ubiquitin chain assembly at Htt-polyQ aggregates, the interactive surface of misfolded Huntingtin species is shielded from unwanted interactions, such as the sequestration of low complexity domain-containing transcription factors that causes transcriptional dysregulation in Huntington's disease. Moreover, LUBAC facilitates proteasomal degradation of misfolded Htt-polyQ species in a p97/VCP-dependent manner (van Well et al., 2019).

Interestingly, a *Drosophila* ortholog of HOIP termed LUBEL (linear ubiquitin E3 ligase) is involved in the heat shock response in flies (Asaoka et al., 2016). Flies expressing catalytically inactive LUBEL mutants show climbing defects and reduced survival upon heat stress, which supports a role of linear ubiquitination in protein quality control.

### Heterotypic Ubiquitin Chains Implicating M1 Linkage

The formation of heterotypic ubiquitin chains strongly diversifies the structure and hence the functional impact of polyubiquitin chains (Haakonsen and Rape, 2019). Heterotypic chains contain more than one linkage type, resulting in mixed or branched ubiquitin chains. In mixed chains, the ubiquitin molecules are connected by different linkage types but each subunit is connected via a lysine or the N-terminal methionine to only one other ubiquitin molecule. In branched chains, at least one ubiquitin subunit is linked to two or even more ubiquitin molecules, which may result in highly complex chain architectures. The mechanisms underlying heterotypic chain formation have not been uncovered in detail yet, but it is emerging that ubiquitin chain initiation, elongation, and branching often requires an intricate cooperation between different E2 and E3 enzymes.

There is increasing evidence for the formation of various branched ubiquitin chains and their specific role in regulating cellular functions. For example, K11/K48-branched chains are characterized by a higher affinity to the proteasome and to p97/VCP and therefore act as a proteasomal priority signal (Meyer and Rape, 2014). In line with such a function, K11/K48-heterotypic chains have been implicated in cell cycle and protein quality control by promoting rapid and efficient proteasomal degradation of mitosis regulators and misfolded cytoplasmic proteins or ERAD substrates (Meyer and Rape, 2014; Yau et al., 2017; Samant et al., 2018; Leto et al., 2019). A structural analysis of branched K11/K48 tri-ubiquitin revealed a unique hydrophobic interdomain interface between the distal ubiquitins that binds the proteasomal receptor Rpn1 with increased affinity (Boughton et al., 2019). Notably, K29/K48- and K48/K63-branched ubiquitin chains can also mediate efficient proteasomal degradation (Kristariyanto et al., 2015; Ohtake et al., 2018).

**Figure 7:**
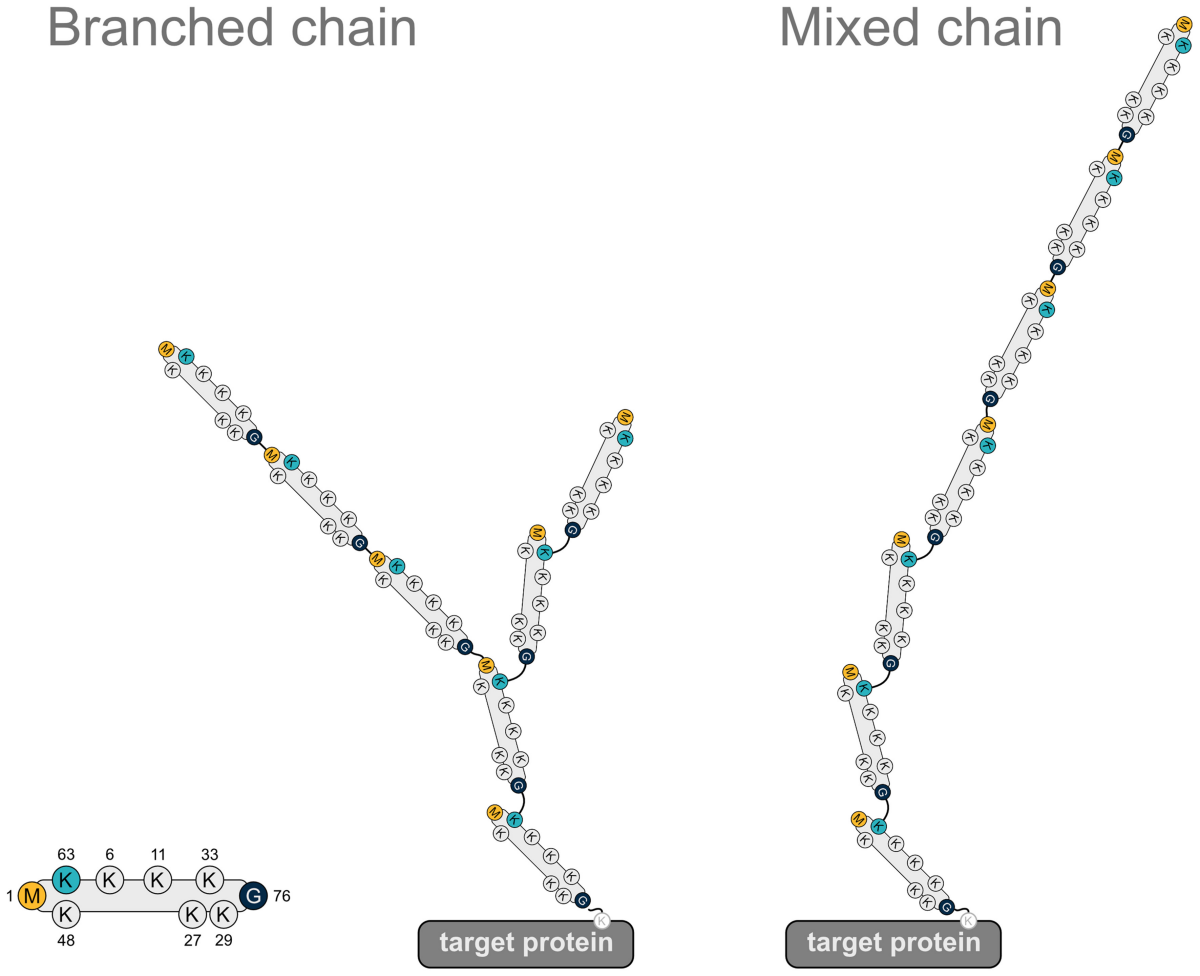
Heterotypic ubiquitin chains implicating M1-linked ubiquitin. Two examples for heterotypic ubiquitin chains containing M1-linked ubiquitin. Linear ubiquitin chains can branch off K63-linked chains via peptide formation between an N-terminal methionine of a ubiquitin molecule within the K63 polyubiquitin and the C-terminal glycine of the incoming ubiquitin (branched chain). Alternatively, the incoming ubiquitin can be added to the N-terminal methionine of the last ubiquitin of the K63-linked chain (mixed chain). G, glycine; K, lysine; M, methionine.

In addition to promoting proteasomal degradation, heterotypic ubiquitin chains play a role in regulating signaling pathways, as has been demonstrated for NF-κB signaling. K48/K63-branched ubiquitin chains generated by TRAF6 (K63) and HUWE1 (K48) in response to IL-1 stimulation amplify NF-κB signaling by protecting from CYLD-mediated hydrolysis of K63-linked ubiquitin (Ohtake et al., 2016). M1-linked ubiquitin chains are also implicated in the formation of heterotypic chains. In fact, most of the M1-linked chains formed upon IL-1 stimulation are covalently attached to K63-linked chains (Emmerich et al., 2013), although the precise topology (mixed or branched, Figure 7) has not been elucidated so far. When K63-linked ubiquitination is inhibited by the deletion of the E2 complex Ubc13-Uev1a, IL-1-induced formation of M1-linked ubiquitin is also strongly reduced, suggesting that K63 ubiquitination is a prerequisite for the formation of M1 ubiquitin chains (Emmerich et al., 2013). In addition to IL-1 signaling, heterotypic M1/K63 ubiquitin chains have been identified upon activation of TNFR1, TLR3, and NOD1 receptors, suggesting that the formation of these hybrid chains is a general feature in innate immune signaling implicating LUBAC (Emmerich et al., 2016). HOIP interacts with K63-linked ubiquitin via its NZF domains, which presumably favors the generation of heterotypic M1/K63 chains (Haas et al., 2009; Emmerich et al., 2013). From a functional perspective, M1/K63 ubiquitin heterotypic chains could act as a platform to co-recruit and concentrate interacting proteins that specifically bind to either K63-linked ubiquitin (such as TAB2 and TAB3 of the TAK1 complex) or M1-linked ubiquitin (such as NEMO of the IKK complex), thereby increasing the efficiency of IKK complex activation (Zhang et al., 2014). It is also conceivable that co-recruitment of regulatory proteins to M1/K63 heterotypic ubiquitin chains helps to fine-tune signaling events in a spatio-temporal manner. In support of this notion, branched M1/K63-linked ubiquitin chains formed upon TNF stimulation inhibit disassembly of K63-linked polyubiquitin by A20 and thus preserve active signaling complexes (Wertz et al., 2015).

## Detection of Linear Chains

### Antibodies

The development of chain-specific antibodies by Newton et al. allows the detection of specific chain topologies using western blotting techniques and has been extended by antibodies developed by other groups (Newton et al., 2008; Matsumoto et al., 2012; Sasaki et al., 2013; Nakayama et al., 2019). The antibodies are raised to specifically recognize the special topology of the ubiquitin chain linkage and can be used for the detection of the chains in western blots, immunocytochemistry and immunohistochemistry (reviewed in van Wijk et al., 2019).

### Tandem Ubiquitin Binding Entities (TUBEs)

Ubiquitin signaling is detected by sets of specific reader molecules. These proteins are able to detect besides the position of the ubiquitination also the topology of the chain. The interaction of the ubiquitin signal reader with the ubiquitin chain is mediated by ubiquitin-interacting motifs in these proteins (Watkins et al., 1993; Bertolaet et al., 2001; Wilkinson et al., 2001; Zhang et al., 2008; Rahighi et al., 2009). By fusing several of these interaction motifs into a new detection molecule, a TUBE is created (Hjerpe and Rodríguez, 2008; Hjerpe et al., 2009). The specificity of the single ubiquitin interaction motif is enhanced by the combination and can then be used for enrichment strategies or a far-western blot experiment.

### Targeted Proteomics

The rapid development of proteomics in the last decade positioned mass spectrometry-based proteomics (discovery proteomics) as the default technique for the detection of several thousand proteins in a single experiment. Parallel to the development of discovery proteomics a second technique for the analysis of samples has been developed. This technology, targeted proteomics, is focussed on the quantification of a specific set of proteins instead of the identification of as many proteins as possible. Here, a specific set of proteins is selected before the measurement and key peptides for the selected proteins are used. By focussing the measurement on a set of key peptides the measurement gains sensitivity, thus allowing the detection of very small amounts of proteins in the sample. The continuous nature of the measurement ensures that the selected peptides will be detected in all samples, if they are present, and avoid the random selection issues that are associated with a shotgun measurement which are responsible for the generation of missing data points. The disadvantage of the method is the preselection of peptides as it does not allow the detection of any other protein than the preselected ones.

### Selected and Parallel Reaction Monitoring (SRM/PRM)

**Figure 8:**
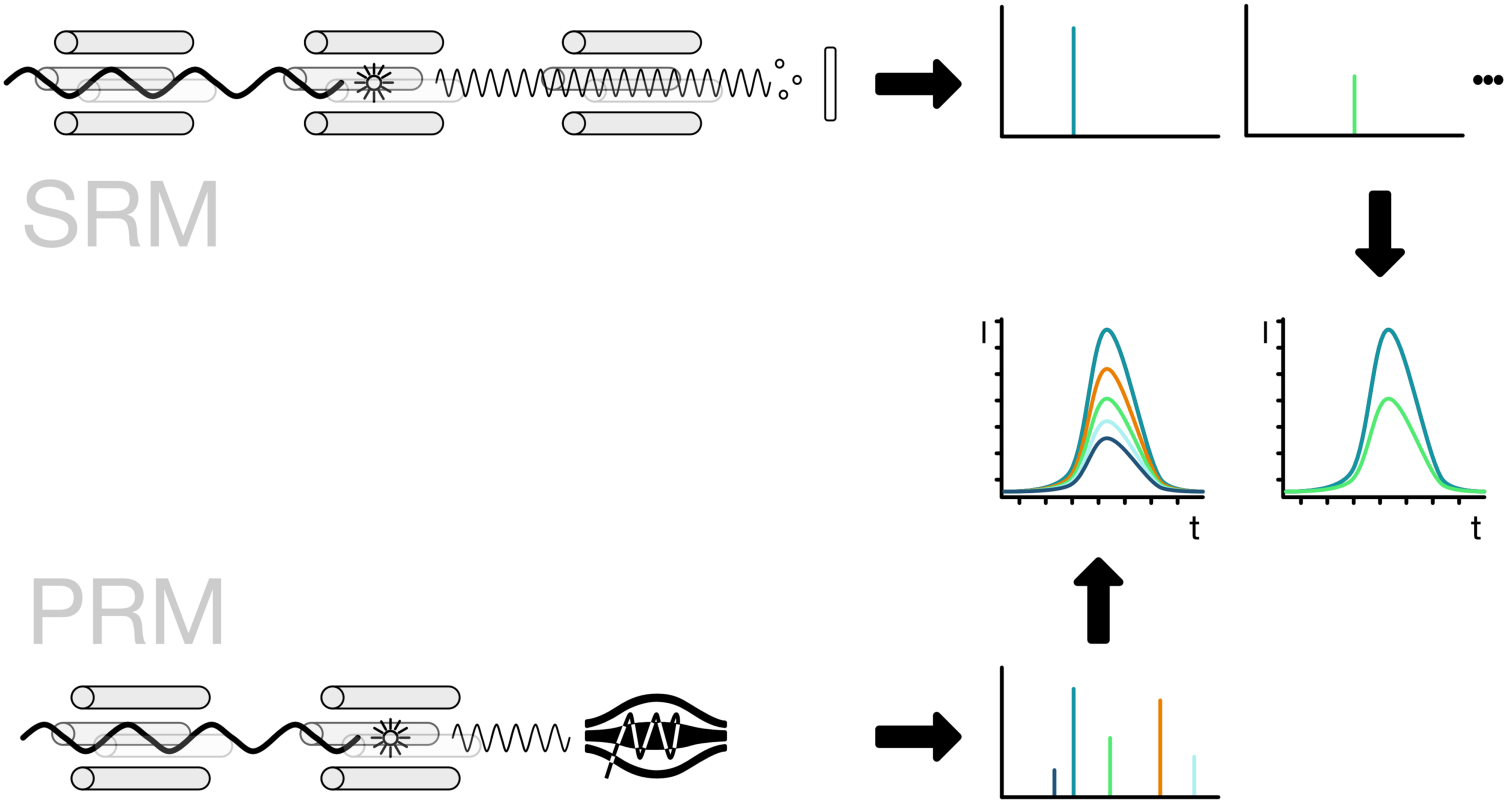
Selected and parallel reaction monitoring (SRM, PRM). The preferred method for the detection and quantification of ubiquitin chains is the use of SRM and PRM. The SRM method is bound to triple-quadrupole mass spectrometers indicated by four rods. The first quadrupole is used for the selection of ions, the second one for the fragmentation of the selected ionized peptide and the third one for the selection of a specific fragment ion. The fragment ions have to be selected one after the other in order to get a measurement for each of them. The PRM method replaces the last step with a scan of all ions in a high-resolution detector like an orbitrap. The selection of the ions is done *in silico*, so the best ions that show no interference can be selected without re-acquiring the spectra.

The implementation of the detection method is usually linked to the use of either triple-quadrupole or Q-orbitrap mass spectrometers. For the specific detection of a peptide, the mass spectrometer has to filter for the full mass of the peptide. The selected peptide is then broken down into fragments by collision-induced fragmentation and key fragment masses are selected for detection. For SRM the selection of the precursor mass is done by the first quadrupole, the fragmentation in the second and the third is selecting specific fragments one after the other. The use of PRM allows the parallel measurement of all peptide fragments for a given mass. For increased sensitivity, the quantification is then based on a smaller number of fragments that are selected to avoid interference from co-selected peptides. Usually, the PRM method employs a high-resolution mass spectrometer thus allowing to further reduce the influence of interfering fragments by applying a strong selection based on the high-resolution measurement (Figure 8; Lange et al., 2008; Mirzaei et al., 2010; Ordureau et al., 2015; Bourmaud et al., 2016).

The identification of the right fragments across a chromatographic separation depends on the elution time and the precursor/fragment pairs. To ensure the right elution points in the gradient, the use of isotope-labeled standards is recommended. Here the same peptide that is monitored using SRM or PRM is chemically synthesized using an isotope-labeled amino acid. Since the light and heavy peptides are chemically identical the peptides co-elute. The quantification of the peptide can then be done between different runs, given the chromatographic setup is stable enough for a comparison. If the isotope-labeled standard peptide is spiked in at a known concentration it can serve as a standard for absolute quantification of the light counterpart.

### Measurement of the Ubiquitin Chain Topology

Post-translational modification by ubiquitin occurs on lysine side chains or the N-terminus of proteins. Here a conjugation cascade connects the C-terminus of ubiquitin to the ε-amino group of lysines or the N-terminal amino group. The target protein can be a protein that is regulated by ubiquitination or ubiquitin itself, forming ubiquitin chains.

**Figure 9:**
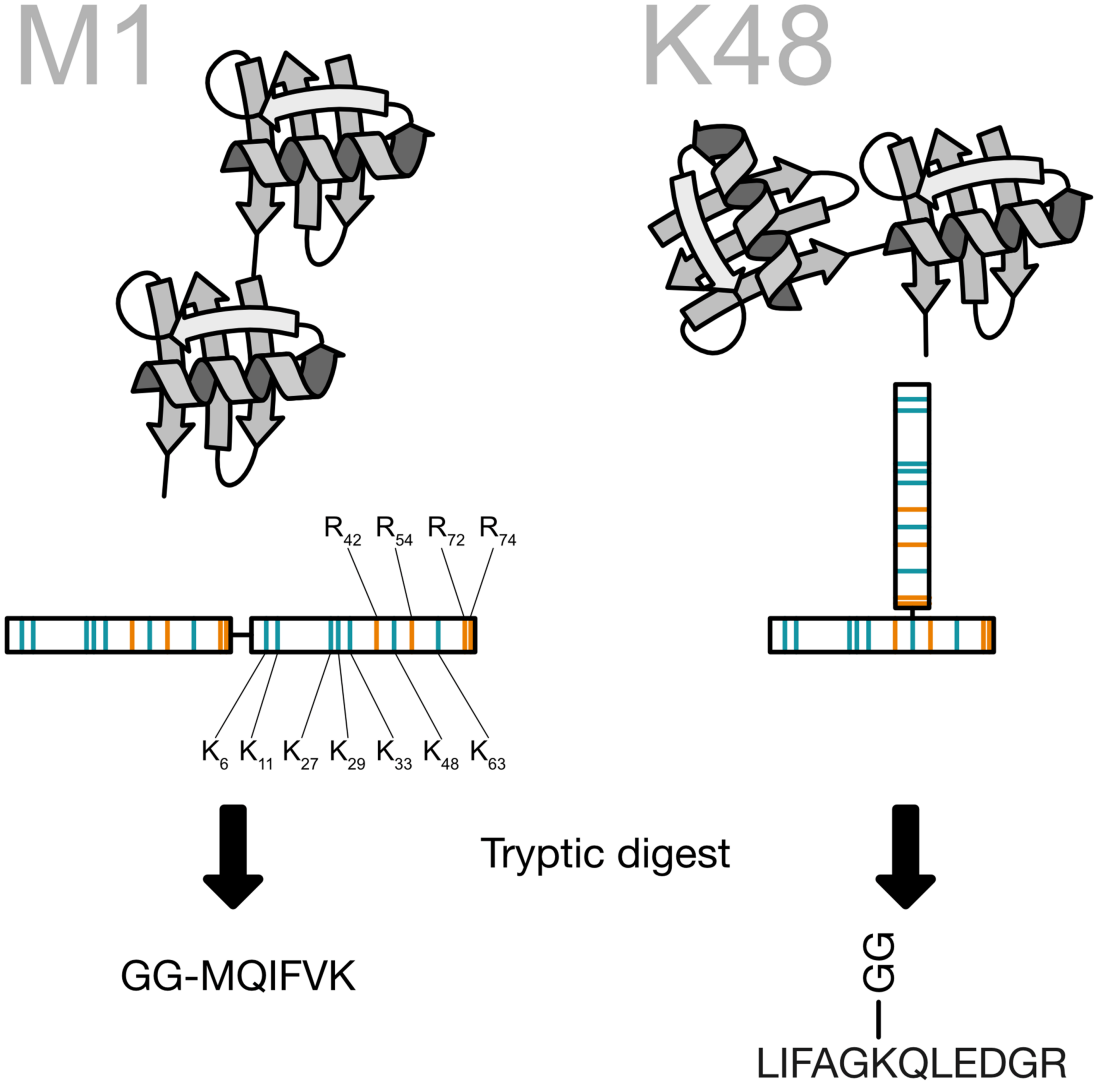
Generation of the ubiquitin chain specific peptides. The C-terminus of ubiquitin is bound to a lysine side chain of the previous ubiquitin. Ubiquitin contains a number of arginine residues that are recognized by the protease trypsin. By cutting after arginine 74 the last two amino acids of ubiquitin are remaining on the lysine side chain and create a peptide, which carries two glycines on the ε-amino group of the lysine. This prevents at the same time a digestion of the modified lysine. All key peptides for ubiquitin chains carry the two glycine residues on a specific lysine side chain except for linear ubiquitin, where the ubiquitin is fused head-to-tail. This particular key peptide carries the two glycine residues on the N-terminus.

Ubiquitin chain topology analysis takes advantage of this unique shape. When a ubiquitin chain is digested with the endoproteinase trypsin key peptides are generated that carry two glycine residues on the side chain. These two remnant residues are coming from the -RGG C-terminus of the next ubiquitin in the chain and are cut off by trypsin during the generation of the peptides (Peng and Gygi, 2001). For the N-terminal ubiquitin fusion, a signature peptide starting with the GG and continuing with the N-terminus of ubiquitin is created by the tryptic digestion. These signature peptides are then used as surrogates for the presence of ubiquitin chains and can be quantified using different targeted proteomics techniques (Figure 9). Recently, the Komander group created a viral protease-derived recombinant protease, which recognizes the C-terminus of ubiquitin (Swatek et al., 2018, 2019). The cleavage of the ubiquitin chains occurs after arginine 74 and leaves the GG-remnant on the lysine side chain of ubiquitin or the substrate protein. The analysis of ubiquitin by intact mass spectrometry revealed ubiquitin molecules decorated with several GG-remnants. Quantification of this ubiquitin population shows that 10–20% of the ubiquitin chains are branched (Swatek et al., 2019).

Studies using middle-down proteomics, which is based on a partial tryptic digestion of ubiquitin chains, were able to elucidate the chain length and branching of the polyubiquitin chain (Xu and Peng, 2008; Valkevich et al., 2014; Rana et al., 2017).

A different approach has been developed by Tsuchiya et al. using a TUBE construct with a tryptic digest for the mass spectrometric analysis (trypsin-resistant TUBE, TR-TUBE). The TR-TUBE binds the polyubiquitin chain and is used to pull out the ubiquitinated substrate proteins. In a subsequent tryptic digest the substrate proteins is identified. This allows the distinction of the mono-ubiquitinated proteins from polyubiquitinated ones (Tsuchiya et al., 2018).

### Difficulties of Ubiquitin Chain Topology Detection

Ubiquitin-associated signaling can, like other post-translational signals, be erased by two different mechanisms, the dissociation of the ubiquitin chain from the protein target or the degradation of the protein target. While the degradation of the target protein is mostly associated with K48 and K11 chains (Chau et al., 1989; Williamson et al., 2011), the dissociation of ubiquitin chains by deubiquitinating enzymes can occur on all types of chains. For the detection of degradation-mediating chains, the inhibition of the proteasome as the endpoint of the reaction can lead to stabilization of the chains (Kim et al., 2011; Wagner et al., 2011). For the detection of linear chains, proteasome-associated degradation seems to be less important, but the disassembly reaction of chains can severely impair the detection. For the inhibition of the disassembly reaction, several techniques have been employed. The use of highly denaturing agents during the lysis of the cells, using guanidinium hydrochloride (Lectez et al., 2014) or urea (Peng et al., 2003; Bagola et al., 2013), have been proven effective. Other strategies include the precipitation of proteins prior to the extraction using tri-chloric acid (Ziv et al., 2011) or chemical inhibition which is widely used. Most of the deubiquitinating enzymes belong to the class of cysteine proteases which carry a cysteine in the active center of the enzyme. Alkylating agents specific to sulfhydryl groups can be used to modify the active center and thus inactivate the enzyme. N-ethylmalemide and iodoacetamide are widely used, although it has been shown that iodoacetamide can cause unspecific modification of lysine side chains with the same molecular weight as a double-glycine modification (Nielsen et al., 2008), leading to false detection of ubiquitination sites. Other modifications and the inability of certain proteomic search engines to detect ubiquitination appropriately can lead to false-positive identifications as reviewed in Beaudette et al. (2016).

Ubiquitin has a very stable fold and can easily refold after heat or denaturation using chemical agents. This can pose a significant challenge to the accurate quantification of ubiquitin chains using mass spectrometric techniques. The generation of the ubiquitin peptides is dependent on the accessibility of all potential cleavage sites to the proteases used. If ubiquitin is not completely digested this would lead to a significant underestimation of the ubiquitin-derived peptides. Strategies using a two-step digestion protocol under highly denaturing conditions (8M urea) with a protease like endopeptidase lysC in a first step to degrade the protein into smaller pieces, followed by a dilution step and the digestion with trypsin, as trypsin is not active under highly denaturing conditions (de Godoy et al., 2008). Alternatively, the addition of mild mass-spectrometry-compatible detergents, like RapiGest, can enhance the sensitivity of the detection (Longworth and Dittmar, 2019).

### Advantages and Disadvantages of Using Chain-Specific Antibodies vs. Detection by Mass Spectrometry

The detection of ubiquitin chain topology using antibodies has the obvious advantage of not being dependent on a mass spectrometry laboratory and usually the rapid detection associated with the simple western blot setup. Although the quality of the chain topology-dependent antibodies has improved over time, the quality of the antibody is still dependent on production batches and can vary significantly between batches. The quantification of different chain topologies is difficult and the quantification across different topologies requires high-quality standard to be added to the analysis. The mass spectrometry-based techniques are independent of production batches, but face other challenges. The above-mentioned digestion problem can lead to a significant underestimation of the chains. The determination of ubiquitin topologies is dependent on the detection of a single peptide, which carries the specific modification. Each of the seven characteristic peptides has its own affinity for unspecific absorption to plasticware. In order to prevent the unspecific loss of peptides due to absorption, the use of a carrier (like an *E. coli* digest) has proven effective to prevent losses of the peptides and the spike-in reference peptide (Longworth and Dittmar, 2019). The use of a spike-in reference peptide (Ubi-AQUA) provides the possibility for absolute quantification (Kirkpatrick et al., 2006; Mirzaei et al., 2010; Ordureau et al., 2015). Here special attention to the possibility of losses due to absorption has to be considered, as it can lead to incorrect quantification of the reference standard prior to the spike-in. The quantification of the characteristic peptides can also pose a challenge as some of the peptides show a tendency for the formation of double peaks that are hard to quantify reliably, although changes to the chromatographic setup can minimize the effect (personal observation).

## Author Contributions

All authors listed have made a substantial, direct and intellectual contribution to the work, and approved it for publication.

### Conflict of Interest

The authors declare that the research was conducted in the absence of any commercial or financial relationships that could be construed as a potential conflict of interest.

## Abbreviations

BCL10: B cell leukemia/lymphoma 10
cIAP: cellular inhibitor of apoptosis protein
CARD: caspase recruitment domain
CYLD: cylindromatosis lysine 63 deubiquitinase
DLBCL: diffuse large B cell lymphoma
ERAD: endoplasmic reticulum-associated degradation
HOIL-1: haem-oxidized iron-regulatory protein 2 ubiquitin ligase-1
HOIP: HOIL-1-interacting protein
HUWE1: HECT, UBA and WWE domain containing E3 ubiquitin protein ligase 1
IKK: IκB kinase complex
IRAK: interleukin 1 receptor associated kinase
IRP2: iron regulatory protein 2
KLN1: kinetochore null protein 1
LDD: linear ubiquitin chain determining domain
LPS: lipopolysaccharide
LUBAC: linear ubiquitin chain assembly complex
LUBEL: linear ubiquitin E3 ligase
MALT1: mucosa-associated lymphoid tissue protein-1
MLKL: mixed lineage kinase domain-like
MyD88: myeloid differentiation primary response gene 88
NEMO: NF-κB essential modifier
NF-κB: nuclear factor of kappa light polypeptide gene enhancer in B-cells
NLRP3: NLR family pyrin domain containing 3
NOD: nucleotide binding oligomerization domain containing
ORAS: OTULIN-related inflammatory syndrome
RIG-I: retinoic acid-inducible gene-I
RING: really interesting new gene
RIPK: receptor-interacting serine/threonine-protein kinase
RBR: RING-in-between-RING
SHARPIN: Shank-associated RH domain-interacting protein
SNP: single nucleotide polymorphism
SPATA2: spermatogenesis-associated protein 2
UBA: ubiquitin-associated
UBD: ubiquitin-binding domain
UBAN: ubiquitin-binding domain in ABIN proteins and NEMO
UBL: ubiquitin-like
PIM: PUB-interacting motif
PUB: PNGase/UBA or UBX-containing proteins
TAB: TAK1-binding protein
TAK1: transforming growth factor-β-activated kinase 1
TBK1: TANK binding kinase
TLR: Toll-like receptor
TNF: tumor necrosis factor
TNFR: TNF receptor
TRAF: TNF receptor-associated factor
TRAIL: TNF-related apoptosis-inducing ligand
VCP/p97: valosin-containing protein
XIAP: X-linked inhibitor of apoptosis.
